# Superintendent Worsham on Insanity

**Published:** 1908-05

**Authors:** 


### Superintendent Worsham on Insanity.

The following "interview" with Dr. Worsham appeared in the *Houston Post* of March 24, 1908:

Dr. B. M. Worsham, of Austin, Superintendent of the Texas State Insane Asylum, and one of the best known alienists in the country, is in favor of a law which would place restrictions upon the marriage of persons in whom there is the least taint of insanity. Paradoxical as it may seem, Dr. Worsham, who was at the Rice yesterday, believes that the advancement of science to such a degree that the most acute cases of insanity are cured is the cause of the increasing ratio of insanity, because former insane patients marry, and have children who are defectives.

"Fifty years ago," said Dr. Worsham, "there was absolutely no cure for insanity, and death was the only relief which came to the unfortunate who lost his reason. With the progress of science the most acute cases of insanity are cured, and out of the various asylums in Texas every year there go at least 200 men and women cured of this disease.

"Most of these persons marry. I believe that at the very lowest estimate there are fully 200 children born in Texas every year whose fathers or mothers have at one time been inmates of an asylum of this State. While these men and women who are sent from the asylum as cured are cured, there is still that insane temperament which is transmitted to the progeny. Insanity itself is not transmitted to the offspring, but it is this tendency which brings about insanity.

"And so, while science is curing insanity, the lack of a proper statute which would place restrictions upon the marriage of those who have the insane temperament, although they have been cured in an asylum, causes the percentage of insane persons to the population to be constantly growing. I don't know just how it could be accomplished, but there is necessity for something along this line for the benefit of humanity.

"The population of the Austin asylum is 1200, which is its full capacity. Within the next sixty days a new building will be completed for the use of negro patients. This will permit us to increase the number of negroes there from 200 to 400. There is a demand for these extra accommodations and for more besides, because the jails of Texas have many negroes waiting to be transferred to the asylum as soon as there is room for them there."

Dr. Worsham has been an alienist in the service of the State for eighteen years. He was appointed from Ellis county as first assistant superintendent at Austin. He was there four years, and then became superintendent of the Southwestern Insane Asylum at San Antonio. After eighteen months' service there, he was made superintendent of the Austin institution.

Ethan Allen Hitchcock, former Secretary of the Interior, in whose department the insane asylums of the government are, declared that the institution at Austin was much finer in every respect than any belonging to the Federal government; in fact, was superior in every way to any insane asylum he had ever inspected. That was in 1901, when he was a member of the party which accompanied the late President McKinley on his tour of Texas.

This article elicited a most remarkable letter to Dr. Worsham from a lady who concealed her identity under the appropriate

*nom de plume* of "Proserpine," Mrs. Pluto. From her account, she had "been there." I commit no breach when I publish it with Dr. Worsham's consent, and regret I can not publish it in full. Some of it is unprintable, even in a medical journal. If "Mrs. Pluto" will reveal her identity, Dr. Worsham will be pleased to discuss the subject further with her through the TEXAS MEDICAL JOURNAL. Here follows the letter:

A REMARKABLE LETTER.—AS A WOMAN SEES IT.

HOUSTON, TEXAS, April —, 1908.

*Dr. B. M. Worsham, Superintendent Insane Asylum, Austin, Texas.*

DEAR SIR: Reading your recent views anent increasing insanity, I am moved to dare embezzling a part of your valued reserve time, necessary to peruse this somewhat detailed communication. It strikes me the world of noble men who minister to the sufferings of poor, stricken humanity, too seldom seek for the cause of the ills they would heal, and aim only at effects. Please reflect; I'm not speaking one word in this writing with the idea of either criticizing or antagonizing, but simply and solely in the hope of directing attention where alone it can be made effective in dealing with a mind diseased. Insanity among women is due in 99 per cent of its cases to lack of tender, affectionate love bestowed by the husband, and among men by the excessive waste of the vital forces as represented through the seminal fluids.

This first paragraph may combine both exordium and statement, but let me now state my personal knowledge of the proposition. I was born of parents wholly ignorant of aught save the *fact* of bodily being and its provision. My parents were wedded barely nine months when a son was born; just eighteen months later I opened my eyes to life. Twenty-one months afterward another son came, and at that rate the family increased till nine children ran about the small farm almost naked and often hungry for the barest necessities. * * * I was ushered into mortal existence heart-hungry. I wanted, aye, *needed* love. As I grew up the holy angels alone knew the awful, the agonizing hunger from which I suffered for this heritage of every child, the tenderly expressed love of its parents. Often and often I sat gazing at them, and felt that if only either of them would take me in their arms and give me just one sweet kiss, one endearing embrace, it would transform earth into heaven for me; but neither of them ever gave me such caress until the occasion of my wedding, when they both kissed me good-bye.

Now, I had, in a sense, been driven to wed in the search for this same affection. It seemed too often that I should go mad unless this craving was met in some way. But little past fifteen years of age, I accepted the suit of a man about twelve years my senior. His demonstrations before marriage led me to think my *heart*-longing would, at least, be gratified. Sexual desire I knew nothing of, nor, to this day, have I ever been led to know its nature. I believe that, had my affectional nature been met, and my natural propensities cultivated, the human fruitage of womanhood had been my portion; but this man the world called my husband, because a human and not a divine code had been pronounced, * * * no sooner had I been left alone with him than he assaulted me and committed the most dastardly rape on my person a man could be guilty of perpetrating. The days that followed were one round of vulgar exhibitions and demands for the most offensive sacrifices on my part. I demurred and he roughly demanded, "What d'you s'pose I married you fur? That's all men want," etc. I was untaught as to the conditions prevailing in the wedded estate. I had no means of knowing whether all women were required to descend to like humiliation or not; but, judging from what my own mother had told me of my father's doings, I was half convinced my husband (?) was right. I bore the condition—in my young ignorance knew no better—for three years. A child was born, meantime, and the beast did nothing for our support; lay about saloons and was drunk half the time. Explaining to his associates that "his wife was no wife to him," and that, of course, commanded their sympathy. I took my babe, at last, and fled from him; for I would have numbered one of the unfortunates over whom you now have charge had I been bound to remain in such torment. All this time, the dread longing for affection was increasing instead of diminishing; for sexual congress was *not* what my nature needed.

Not supposing, however, that all men were wholly alike, some years later I again contracted matrimonial rites. The man in this case was an educated, refined gentleman, as the word goes. He never spoke a vulgar word in my hearing, nor was guilty of an offensive act; but he knew no more of affection—the kind my heart ached and hungered and longer for—than though he had been made of rock. He was not specially intemperate, did not seek sexual congress so very frequently, nor in any objective way did he ever offend my sensibilities; but never in all the years we passed together—fourteen—did he volunteer one single expression of that tenderness *I needed.* Whenever he did offer me a caress, I knew the sex impulse was its cause, for it was always followed by such demand, and then loving expressions ended until next time the same nature was to be met. When I asked him why he never offered me a caress except when he wanted such service, he replied, "I don't think about it." Fatal admission! It proved that no love-impulses dwelt in his heart, and only sexual ones in his fleshly nature. The fact extinguished the love fires—what there had been of them—in my nature. He, caring only for a woman who promoted the sexual impluses, soon sought another woman. I truly loved the higher self of him, and never in all our lives had I failed to shower the full affection of my heart upon him. Whenever engaged at household duties and they led me past where he sat reading, or any such opportunity offered, I never failed to hastily press a loving kiss on his forehead, stop and stroke his hair, or in some way show the love of my heart for him. Never, though, never once, did he respond to such embraces on my part. Will add just here that my entire nature is one bundle of affection. I can not live anywhere, with children, my own sex, animal pets, or in any manner where affection is permissible at all, without a constant expression of my heartfelt love for life in its highest and most refined forms; but debauchery, the descent to animalized planes for the revelry of the senses in the depths of vulgarity and debauched life, I *can not* endure. This second husband finally deserted me for a red-light woman, an ignorant, uncultured female, but one who could—at least, did—supply his fleshly needs without demand on his heart,—at least, so I concluded, though I have no means of knowing just how her inner selfhood is organized.

My friends who understood me best, both men and women, often assure me: "You haven't met the right man yet," etc. Well, I rather objected to trying them all in order to "find the right one." but I did, finally, wed again. This time a man of mature years, supposing that time would serve to transmute the baser factors of man's nature into finer forces, but, alas! he was a worse beast than the first, in that he added an educated power to the lesser strength of ignorance. Widely traveled and experienced, he lived only for descent into the more insiduous debaucheries and suggested such awful * * * that I would have fled from him in two days but for the opinion of my friends and the social world in which I moved, added to the fact of two previous failures (?) matrimonially, from which the world's judges would deduce that, since I was unable to live with any man, the cault must be mine. Very logical, indeed!

Back to my primary proposition anent insanity: I base my conclusion not upon my own knowledge alone, but my sphere in life has brought into my confidence a large number of other women who have given me their confidence, thereby seeking some solution of the dread problem of matrimonial union; and in a very large percentage of instances I find their worst trials are just what mine were, *i. e.*, lack of affection and an over-supply of lust. Many, many of my sister women have said to me, with tears streaming from horrified eyes, "I can not endure my life. I feel I shall go mad," etc. Woman's nature needs love, not lustful desire unfortified by aught save selfishness, and, needing this, they perish, in a soul sense, without it as a plant dies from want of refreshing raindrops, and is scorched and withered and destroyed by the blazing fires of the sun, unmodified by refreshing forces from other of nature's sources.

Now, while you, the medical world—and remember, I am not speaking with disrespect of the fraternity—why don't you men seek for the cause of insanity, and strive to ameliorate the conditions which force its increase? Where and when do we hear of virtue among men being taught? On the contrary, it is taught—and by physicians—that sexual congress is essential to the health of men. * * * Forced congress is the indirect cause of insanity in both sexes. Men are depleted of their most potent vital force and women are made the victims while their very souls sicken with disgust, and then the world wonders at increasing insanity!

Suppose there appeared among the people of a civilized community a species of wild beast that devoured, not the souls, but only the bodies of women and children. Would not the entire machinery of civilization be swiftly set into action for the destruction of such wild beast? Yet what do we see as a factor of civilization—Christian civilization, too—when admittedly the bodies and souls of women and children are daily sacrificed to the god Lust (Moloch). Not only is no effort exerted to prevent the destruction, but, as I have pointed out, the condition is fortified by men in every possible fashion. Houses of prostitution are licensed; the statute books are blotted by a code providing an "age of consent," a shame to mankind, since the supply for these houses, as shown by Chas. Crittenden in his recent address in Houston, is at the rate of 60,000 girls annually!

I repeat, these facts are the direct and indirect causes of increasing insanity; for the matter of that, I believe them to be the cause of insanity primarily. I believe that were men taught to live above the base animal plane, in the wedded estate, dispensing that sweet tenderness the *heart* needs, and without which it dies, their own joys in being and delights in living would not only be heightened to an inconceivable degree, but the fructifying force of love would then promote a sanative condition both physically and psychically whence not only human happiness would spring to brighten the lives of humankind, but that higher, that heavenly happiness whence the soul-being issues would be so regenerated that angles would be born into the earth-life, dowered with strength and love and good, bearing no taint of those dread weaknesses that are the seeds of insanity sown in the mother's womb under the impulse of lust. * * *

Sexuality is a manifestation of divine forces on this plane, and they are meant to lead to higher heights. They *would* so lead, but, perverted, they destroy the fountain of objective being, *i. e.,* motherhood. Motherhood, this heavenly fountain, is thus polluted with over-feeding of lust and under-feeding of love, and the root of insanity is planted in the garden of life. Its fruitings are filling such institutions as you preside over, cause the State and nation dreadful losses annually through crime and failure to reach humanity's highest estate; and all through an improper adjustment of laws which are, I believe in general, ignorantly violated, but none the less fatally because ignorantly violated.

Set about *enforcing* restraint,—a better term is "control,"— of the sex forces in men, and there will be less of insanity to deal with. Instead of promoting in every possible way the privileges men deem so very essential to their *health,* castrate, if necessary, men unable to exercise self-control. That would be far more humane than allowing them to run uncontrolled until they land in the asylum and must remain in bodily as well as mental restraint.

The subject is endless, and I'll trouble you no further at this time, but my labors along this line are not finished. I've had my schooling,—indeed, I may well claim a diploma in the school,—and feel I should prove false to my education did I not use it for the amelioration of my own sex, who are the greatest sufferers, and next for the race in general. If you care to hear from me further, you may send a letter to the *Post* to be published in "Letters from the People" on each Monday morning. I'm impressed, however, that you, in kind with men in general, will resent all interference with "marital rights."

Very much in earnest,

"PROSERPINE" (MRS. PLUTOQ.

